# ASSOCIATION OF CHILDHOOD EXPOSURE TO SCHOOL RACIAL SEGREGATION WITH LATE-LIFE COGNITION AMONG AMERICAN ADULTS

**DOI:** 10.1093/geroni/igad104.1233

**Published:** 2023-12-21

**Authors:** Zhuoer Lin, Yi Wang, Thomas Gill, Xi Chen

**Affiliations:** Yale University, New Haven, Connecticut, United States; Yale University, New Haven, Connecticut, United States; Yale School of Medicine, New Haven, Connecticut, United States; Yale University, New Haven, Connecticut, United States

## Abstract

Racial segregation may contribute to enduringly worse health outcomes and aging. Prior research mainly focuses on residential segregation, yet the long-term effects of school segregation and the effects for different racial/ethnic groups are largely unknown. Linking measures of primary school segregation using administrative data with a nationally representative population survey in the US, we examine how childhood exposure to school racial segregation shapes the late-life cognitive function. 23,752 non-Hispanic White (White), 6,364 non-Hispanic Black (Black) American adults aged 50 and older were identified from the Health and Retirement Study; and state-level school dissimilarity indexes in late 1960s were linked to participants’ latest wave of cognitive assessment using childhood residence. Multivariate regression analyses demonstrated that exposure to White-Black school segregation had strong, negative effects on cognitive outcomes for both White and Black participants. One standard deviation increase in dissimilarity index was associated with lower cognitive score (White: = -0.16 [95%CI, -0.22, -0.11]; Black: = -0.42 [95%CI, -0.53, -0.30]) and more cognitive impairment (White: Odds Ratio (OR) = 1.10 [95%CI, 1.06, 1.13]; Black: OR = 1.21 [95%CI, 1.14, 1.27]) adjusting for age and sex. Educational attainment and other socioeconomic characteristics explained most associations (40-70%), and the effects remained significant even after accounting for a rich set of late-life factors. Overall, these findings suggest that childhood exposure to school segregation has long-lasting effects on late-life cognition for both White and Black Americans. Educational policy and reform should be promoted to reduce school racial segregation within and between school districts to address health inequities.

